# Market Powers Predict Reciprocal Grooming in Golden Snub-Nosed Monkeys (*Rhinopithecus roxellana*)

**DOI:** 10.1371/journal.pone.0036802

**Published:** 2012-05-09

**Authors:** Wei Wei, Xiao-Guang Qi, Song-Tao Guo, Da-Peng Zhao, Peng Zhang, Kang Huang, Bao-Guo Li

**Affiliations:** 1 Key Laboratory of Resource Biology and Biotechnology in Western China, College of Life Sciences, Northwest University, Xi'an, China; 2 School of Sociology and Anthropology, Sun Yat-Sen University, Guangzhou, China; 3 Institute of Zoology, Shaanxi Academy of Sciences, Xi'an, China; University of Arizona, United States of America

## Abstract

Social grooming is a common form of affiliative behavior in primates. Biological market theory suggests that grooming can be traded either for grooming or other social commodities and services. When no other services are exchanged, grooming is predicted to be approximately reciprocated within a dyad. In contrast, the amount of reciprocal grooming should decrease as other offered services increase. We studied grooming patterns between polygamous male and female in golden snub-nosed monkeys (*Rhinopithecus roxellana*) from the Qinling Mountains of central China and found that about 29.7% of grooming bouts were reciprocated. However, the durations of grooming bouts offered and returned was asymmetrical within dyads. In bisexual dyads, more grooming was initiated by females than males, which became more pronounced as the number of females per one-male unit increased. The rate of copulation per day for each female was positively correlated with the total duration of grooming time females invested in males.. Females without an infant (non-mothers) directed more grooming towards females with an infant (mothers) and were significantly more likely to be non-reciprocated. There was a significant negative relationship between non-mother and mother grooming duration and the rate of infants per female in each one-male unit. High-ranking females also received more grooming from low-ranking females than vice versa. The rate of food-related aggressive interactions was per day for low-ranking females was negatively correlated with the duration of grooming that low-ranking females gave to high-ranking females. Our results showed that grooming reciprocation in *R. roxellana* was discrepancy. This investment-reciprocity rate could be explained by the exchange of other social services in lieu of grooming.

## Introduction

Grooming is a common form of affiliative behavior in various mammalian species of ungulates, rodents, and carnivores, and is especially important for species with complex social systems such as primates [1], [2]. Social primates devote a significant proportion of their time (2%–5%) to exchanging grooming with their conspecifics [3], [4]. A number of theories have been proposed to account for this time investment. The ‘hygienic hypothesis’ states that the primary function of grooming is to assist in removing ectoparasites from body areas that the beneficiary cannot easily reach [5], [6]. However, this hypothesis does not satisfactorily explain grooming on body areas accessible to the beneficiary [7], [8]. In addition, grooming appears to increase psychological and physiological well-being through the release of *β*-endorphine [9], [10] and the decrease in heart rate [11], [12]. Furthermore, social functions of grooming have been widely suggested as a way to establish harmonious relationships between group members and for the maintenance of social affinity [13], [14].

Concerned with the costs associated with grooming, kin selection theory has been used to explain the disproportionate amount of grooming among relatives [15], [16]. However, the kin-selection hypothesis does not adequately explain grooming in non-kin dyads. Trivers [17] proposed that grooming among non-kin individuals represents a form of reciprocal altruism. This hypothesis assumes that altruistic behavior is favored if individuals benefit from the reciprocal interaction, and is employed for the maintenance of social bonds and coalitionary support [14], [18]. Reciprocal altruism offers a process to explain the evolution of altruistic behaviors among unrelated animals (see [19] meta-analysis). Based on these progresses, more recent biological market theory [20], [21] indicates that social animals have a great deal of potential partners to choose from, individuals could exchange valuable acts to obtain commodities or partners corporation which they have limited access and demand to gain. Biological market theory emphasizes the varying balance between giving and receiving due to economic forces such as fluctuating demand/supply ratios [22], [23]. As the most common social behavior in primates, grooming can be exchanged either for itself or for other beneficial services with group members. The biological market approach suggests that the decision to do either will depend on individuals standing in the market-place and the commodities they can offer within a social group. [24], [25], [26]. When no other services are being exchanged or demanded, grooming is predicted to be approximately reciprocated within a dyad. However, if partners could offer different services, the amount of returned grooming should decrease as other offered services increase. Thus, grooming could be exchanged for a wide variety of possible services such as coalitional support [15], food [27], [28], tolerance [29], mating opportunities [30], [31], information about reproductive status [31], infant holding [32], [33] or for grooming itself [34]. For group-living non-human primates, research has been conducted on a variety of New World and Old World monkeys. Among Old World species, studies of grooming interactions have been conducted almost entirely on Cercopithecinae (*Macaca fascicularis* [31], [32]; *Macaca fuscata* [29], [34], [35]; *Macaca radiata* [36]; *Papio cynocephalus* [24]). Connor [37] proposed the parceling model of reciprocity altruism: within allogrooming interactions in which partners do not groom each other simultaneously, individuals alternate between giving and receiving grooming with each partner performing approximately as much grooming as they received within each bout. Such a model assumes that when grooming is exchanged for itself, immediate reciprocation would be the best way to avoid being cheated and to obtain equivalent value for their services by grooming partners. Models of reciprocity altruism generate predictions about the distribution of grooming within dyads and within grooming bouts.

The golden snub-nosed monkey (*Rhinopithecus roxellana*) is a rare and endangered species endemic to China. A strict seasonal breeder [38], *R. roxellana* lives in a multi-level social organization [39]. As the most complex social structure in non-human primates, multi-level social systems have been found in hamadryas baboons, gelada baboons and snub-nosed monkeys. Such societies are characterized by individual relationships present as two or more levels within the community. In *R. roxellana*, a polygynous colobine species, the basic social and reproductive unit is the one-male unit, which consists of a single resident male, 4.19±1.69 adult females, 0.59±0.72 subadult individuals, 3.94±2.36 juveniles, and1.74±1.38 infants (mean ± SD, *n* = 8 years). The mating season ranges from September to December with births occurring from March to May [40]. The basic social structure is similar to other colobine species, however, one-male units do not repel each other but usually travel together. Several one-male units assemble to form one large troop consisting of more than 100 individuals [41], [42]. Some one-male units undergo fission-fusion and some females disperse between different one-male units [43]. Before sexual maturity, male offspring leave the one-male unit within which they were born and join all-male bands. Female offspring, however, will stay in the one-male unit within which they were born where they become subadult females. Studies of these three species (hamadryas baboons, gelada baboons and snub-nosed monkeys) have shown that *R. roxellana* clearly differs from the other two species in terms of individual social relationships and dynamics [44]. In the gelada baboon, females within the same one-male unit almost never disperse to other one-male units. These females reproduce in the one-male unit within which they were born. Thus harem females in the same one-male unit form a female kinbond. There is a strict female dominance hierarchy among these related females [45], [46]. In contrast, hamadryas baboon, females disperse frequently across different one-male units and are strangers to each other. The compositions of the harems are unstable and frequently undergo fission-fusion. The single resident male needs to utilize sexual attraction to maintain his one-maleunit [47], [48]. Over half of *R. roxellana* females appear to disperse across one-male units. Sometime they even disperse to different troops. Dispersion among females does not occur frequently. The composition of harem females in each one-male unit is stable for a period of time. However, when the single resident male has been in the one-male unit for more than five years or the number of females in the one-male unit becomes large, the frequency of female dispersion will increase. In addition, some females will disperse together from one one-male unit to another one-male unit [43]. Thus, no strict kin-bond exists among harem females in each one-male unit except for a few sisters or mothers and daughters. To establish social relationships between multiple females and the single resident male, individuals may use grooming as an important behavioral strategy to form cooperative alliances and to gain access to resources [49]. Within this framework, it becomes important to evaluate grooming reciprocity patterns in golden snub-nosed monkeys. If grooming asymmetries reflect asymmetries in the services provided by different group members we predict the following:

### (1) In *R. roxellan*

The time the initiator invests in grooming the recipient will predict the probability of grooming reciprocation, namely whether the recipient will reciprocate or not. Among immediately reciprocated grooming bouts, the amount of time that the initiator grooms the recipient and that the recipient grooms the initiator will positively correlate, representing time matching as predicted by reciprocal altruism.

### (2) In male-female dyads, biological market theory predicts a discrepancy in grooming duration, as males are the limiting resource for females in this polygynous colobine species [49]

Females may use grooming to ensure males tolerate them in close proximity, allowing them to establish a good social relationship with the central male. More grooming should thus be directed from females to males than vice versa. During the mating season, the level of asymmetry will increase when the value of this scarce commodity, access to males, is high. This asymmetry will become more pronounced as the number of females per one-male unit increases. If females thus trade grooming for mating opportunities, a positive relationship between the rate of copulation per day and the total duration of grooming time invested in males for each female should exist.

### (3) As social partners, mothers with infants have a special attraction to non-mothers in primate species

Non-mothers can improve their future offspring's survival by attempting to take care of infants from other females to practice mothering skills [50]. Thus infants may represent a valuable commodity for the innate attraction that females hold for infants [32]. Female *R. roxellana* typically give birth to a single infant once every two years, after a six to seven month gestation. If the previous offspring survives to a weaning age of 5–6 months, the mother will not become pregnant in the next year. Infants are individuals aged from 0–6 months old [40], thus the number of infants per one-male unit is relatively small. Females may become more attractive grooming partners to other females when they have young infants [30]. We predicted grooming to be asymmetric in non-mother vs. mother dyads, with more grooming being directed from non-mother females to mothers than vice versa.

### (4) Subordinates might increase their grooming efforts towards dominants, which is the main quantitative measure used to maintain social relationships

Subordinates may trade grooming for tolerance near resources with dominant individuals. Female *R. roxellana* are ranked in a linear dominance hierarchy which fluctuates over time [43], [51]. The degree of reciprocation should be negatively correlated with rank distance. The rate of food-related aggressive interactions per day directed from high-ranking females to low ranking females should then be negatively correlated with the duration of grooming from low ranking females to high ranking females.

## Methods

All research protocols reported in this manuscript were reviewed and approved by the Chinese Academy of Science. Our research received clearance from and complied with the protocols approved by animal care committees of the Wildlife Protection Society of Shaanxi Province, China (permit number: SX43537ACC). All research reported here adhered to the regulatory requirements of Zhouzhi National Reserve, China, where the study took place, and to the American Society of Primatologists principles for the ethical treatment of primates.

### Study Site

The study was conducted in the Yuhuangmiao region of Zhouzhi National Nature Reserve (ZNNR), which is located on the northern slope of the Qinling Mountains, Shaanxi Province, China. (−108°14′–108°18′E, 33°45′–33°50′N, elevation: 1,400–2,896 m above sea level). This region consists of 52,931 km^2^ of temperate forest. Vegetation types diversify with altitude, consisting of deciduous broadleaf forest from 1,400–2,200 m, coniferous and deciduous broadleaf mixed forest over 2,200 m and coniferous forest above 2,600 m, and the area has a semi-humid montane climate [52]. Average annual rainfall is approximately 894 mm, with a non-frost period of 150 days. The average annual temperature is 6.4°C, with a minimum of −8.3°C in January and a maximum of 21.7°C in July [40]. The golden snub-nosed monkey is the only resident primate species to subsist in this region. Subjected to seasonal food availability, the major components of the golden snub-nosed monkey diet shift around the year and include items such as seeds, buds, leaves, bark, fruits, and lichen [53].

### Study Troop

There are two troops of golden snub-nosed monkey inhabiting the study area, the East Ridge troop (ERT) and the West Ridge troop (WRT), which are separated by the Nancha River. Based on over ten years continuous observation, the WRT was chosen as the troop for this study. The troop is characterized by a multi-level social structure consisting of an all-male band and one or two bands including one-male units. Details of the study troop have been reported previously [42], [43]. The basic social and reproductive one-male unit usually consists of a single resident male, 4.19±1.69 adult females, 0.59±0.72 subadult individuals, 3.94±2.36 juveniles, and 1.74±1.38 infants (mean ± SD). The average number of females with infants per year is 0.49±0.17 (mean ± SD). Six to eight one-male units, on average, form one group, and the group size will fluctuate with the number of one-male units foraging together in different seasons in the Qinling Mountains [42], [43].

The social composition of the focal one-male units in the WRT during our observation period are presented in Table 1. Twenty-six adult individuals (28 females and 6 males) in 6 one-male units were chosen for this study as their kin and non-kin relationships were clearly and precisely recorded over a decade of research. Permission to conduct this study in a legal manner was received from the ZNNR.

**Table 1:** Compositions of the six study one-male units.

one-male unit	JB	FP	RX	PK	BB	JZT
Group size*	14 16	13 17	11 14	11 13	13 17	9 11
Adult males	1	1	1	1	1	1
Mother/Adult females*	1/5 3/6	3/5 2/5	2/4 2/4	1/4 2/4	1/6 3/6	2/3 1/3
percentage of time grooming	14	11	13	9	16	17
percentage of reciprocated bouts	25	26	28	28	26	30
median rank distance between grooming partners	3	3	2	2	3	2

* There were two observation periods: the first row represents the first period (2009.3–2010.1), the second row represents the second period (2010.3–2010.7). Female *R. roxellana* typically give birth to a single infant. Thus, in our observation period the number of mothers equals the number of infants in each one-male unit.

Individual identification was based upon prominent physical characteristics, such as facial contours, body size, pelage coloration, crown hair pattern, scars, evidence of previous injury (physical disabilities), and the shape of granulomatous flanges on both sides of the upper lip [38], [42]. In this study, we focused on two age-sex classes of monkeys, which were defined as follows:

#### (a) Adult males (over 7 years old)

Adult males with conspicuous large bodies covered with extraordinarily long, brilliantly golden guard hairs across the entire dorsum. Body areas, such as the cape area, the dorsum, crown to nape, and arms were deep brown. The granulomatous flanges were obviously visible as were two large upper canines.

#### (b) Adult female

(more than 5 years): Adult females were approximately two thirds the size of adult males. The color of the dorsum, crown to nape, cape area, arms, and outer thighs were brown and changed to a deeper brown with increasing age. The golden guard hairs over the dorsum and cape area were also brilliant but much shorter than those of adult males. Some individuals had very small but visible granulomatous flanges as well. Their breasts and nipples were large and visible.

### Data Collection

Behavioral observations were conducted from March 2009 to July 2010 for a total of 182 days and 964 hr. The behavioral data were collected in the form of focal animal behavioral sampling [54] across a period of three consecutive hours to record patterns of grooming and other social interactions among all individuals residing in the focal one-male unit. Individuals of a one-male unit usually stay within close proximity to one other, so all adult individuals were observable simultaneously for the focal one-male unit. The onsets and terminations of allogrooming interactions by each partner were recorded to the nearest second, along with the identity of the participants. One-male units were studied in an alternating order each day (from 10:00 to 16:00) keeping a distance of between 0.5 and 50 m from the focal animals. If visual contact with the target one-male unit was lost or if most of the one-male unit individuals were lost, a new one-male unit was selected for study and followed for a period of 3 hr. On average, two target one-male units were studied each observation day with each one-male unit being observed for a total of 160 hr.

A grooming bout atarted when one of the two partners initiated the first grooming episode, and ending when the individuals separated from each other, or if no grooming was exchanged for more than 600 s. Episodes were defined as grooming trade-offs within grooming bouts. In a grooming bout, if individual A groomed B and B reciprocated, the bout was composed of two episodes: the episode in which A groomed B and the episode in which B groomed A [55]. Henzi et al. [56] showed that within-bout reciprocation is essential for the maintenance of grooming dyads over time, suggesting that there is something critically important about the capacity to respond to grooming immediately. Given this and the problem of determining *a priori* the period over which to measure responses to non-reciprocated bouts (minutes, hours or days), our analyses were based only on immediately reciprocated bouts.

We recorded copulation behavior by identifying the initiator and receiver. A copulation act involved mounting, including heterosexual genital contact accompanied by intromission and pelvic thrusts [38]. We calculated the rate of copulation per day for each female.

For each agonistic event, we recorded the identity of the initiator and the receiver and the behavioral context in which the interaction occurred. Aggressive behavior (biting, fighting, chasing, threatening, supplanting) and submissive behavior (avoiding, crouching, retreating) were both recorded. We calculated the rate of aggressive interaction events per statistical day for each individual.

### Data Analysis

Weighted logistic regression was conducted to test whether the duration of time the initiator groomed the recipient in the first grooming episode predicted whether the recipient reciprocated or not [57]. Grooming partners were randomly assigned as the initiator and recipient in the regression model in each bout. Thus, there was a theoretical maximum value of *n*(*n*−1) dyads per one-male unit, where *n* was the number of individuals. However, according to Henzi et al. [56], not all individuals are available to groom all other one-male unit potential dyads members. Sample sizes from this study were all smaller than the above-mentioned maximum value. The weight of every bout was defined by the inverse number of bouts in each dyad. Owing to a right-tailed skew in the distribution of the time the initiator groomed the recipient, the variable logarithm was transformed before analysis.

Maximum likelihood estimation was used to avoid pseudoreplication of clusters containing the same animals [57], [58]. For all reciprocated grooming bouts, we conducted a weighted least-squares regression to test the hypothesis that grooming was time matched within these bouts, (i.e. total duration of time that the initiator groomed the recipient predicted the total duration of time that the recipient grommed the initiator). Each observation was weighted as described above. Grooming durations were logarithm transformed and normalized by subtracting the mean then dividing the differences by the standard deviation.

To elucidate overall grooming reciprocity and investigate whether the reciprocation of grooming was approximately equal between partners within dyads, a reciprocity index (*R*) was calculated [26]: 
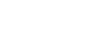
The *R*-index, which is a measure of the symmetry of interactions, is calculated by subtracting the total amount of grooming received by a particular individual from the amount of grooming given by that individual, and dividing the result by the total amount of grooming to correct for sample size. *G*_AB_ is the amount of grooming that individual A gave to individual B, and *G*_BA_ is the amount of grooming given by individual B to individual A. The *R* index ranges from −1 to 1, with positive values indicating that partner A gave more grooming than he/she received, a value of 1 representing complete grooming altruism. Negative values indicate that individual B gave more grooming than he/she received, representing selfishness on the part of A. A value of 0 represents even reciprocity. *R* index results are presented as mean ± SE. We classified the females into different categories. To avoid pseudoreplication of dyads including the same individuals, according to the different trader classes, interactions within a dyad were classified as individuals of A or B separately (Table 2). Independent-Samples T-Tests were used to test differences between categories.

**Table 2:** Categories to which dyads of *R. roxellana* were assigned according to their membership in different ‘trader’ classes.

Dyads Categories	A	B
MF	Males	Females
NM	Non-mother	Mother
NN	Non-mother	Non-mother
HL	High-ranking females	Low-ranking females

Mother: females with new born infant.

Non-mother: females with no new infant; roles were assigned randomly but consistently within dyads.

We conducted a Spearman rank correlation test to examine the relationship between rank distance and degree of reciprocation (the ratio of reciprocation to total grooming), and to compare the distributions of reciprocal and non-reciprocal grooming. The dominance hierarchy for females was determined based on the outcome of decided agonistic events between females [59]. Dominance ranks were assessed on the basis of the direction and amount of occurrences of aggressive and submissive behaviors which were analyzed using the dominance index method [51], [60]. Rank difference was defined as the recipient's dominance rank minus the initiator's dominance rank, with ‘1’ representing the alpha female's rank [26], [51]. Pearson correlation was used to examine the relationship between non-mother to mother grooming duration and the number of infants per female in each one-male unit, the relationship between the rate of copulation per statistical day for each female and the total duration of grooming time females invested in males, and the correlation between the rate of food- related aggressive interactions per statistical day and the duration of grooming given to high ranking females from low ranking females. We conducted chi-square tests to compare the frequencies of aggressive interactions among females during and after mating seasons. The Chi-square goodness of fit test was used to compare the expected duration and the observed duration of grooming that each resident male reciprocated to his harem females. Statistical tests on grooming were all two-tailed. All statistics were performed using the Stata 11/SE software package.

## Results

### Distribution of probability of grooming reciprocation

**Figure 1:**
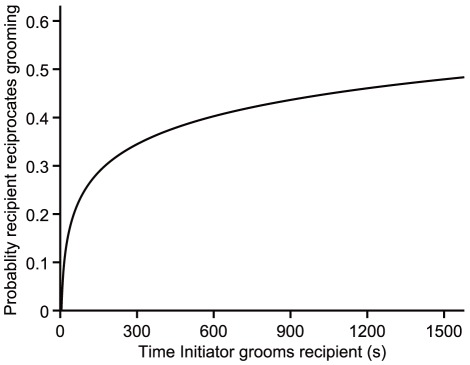
Duration of grooming (s) by the initiator versus probability of grooming reciprocation by the recipient in golden snub-nosed monkeys (*N* = 855 bouts). Within a bout, if the initiator invests longer time in grooming the recipient in the first episode, the probability of the grooming reciprocation deriving from the recipient to the initiator will be higher.

**Figure 2:**
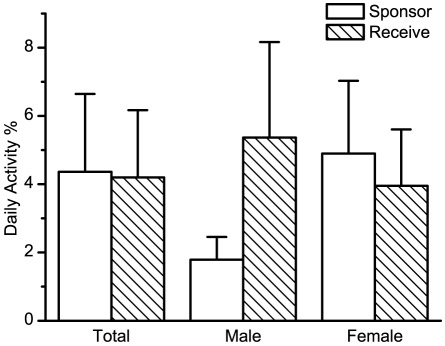
Percentage of given and returned grooming in relation to daily activity in adult individuals. In adult individuals, the given and received grooming accounted for 4.36%±2.28% and 4.19%±1.97% (mean ± SD) of daily activity, respectively. A adult males spent less time in giving grooming than receiving grooming, 1.79%±0.67% and 5.36%±2.80% (mean ± SD), while the adult femalesspent more time giving grooming than receiving, 4.90%±2.13% and 3.95%±1.65% (mean ± SD).

A total of 855 bouts were recorded, with 29.7% of bouts being reciprocal. The time that the grooming bout initiator spent grooming his/her partner was the main factor affecting the probability that the recipient would reciprocate the grooming (logistic regression, *χ* ^2^ = 42.72, *p*<0.0001). If the initiator invested a longer amount of time in grooming the recipient in the first episode, the probability of grooming reciprocation from the recipient to the initiator within that bout was higher. (*b* ± SE = 0.46±0.07, odds ratio = 1.58; Fig. 1). The average duration of a grooming bout was 455.19±48.23 s (mean ± SE, *n* = 855). During the same bout, the average duration of time that initiator groomed receiver was 288±9.52 s and that receiver groomed initiator was 78±5.53 s (mean ± SE, *n* = 855). The grooming took up 4.36%±2.28% (mean ± SD) of daily activity, which was calculated by the sum of grooming time divided by the total observed time for each individual (Fig. 2).

### Time matching within reciprocated grooming bouts

**Figure 3:**
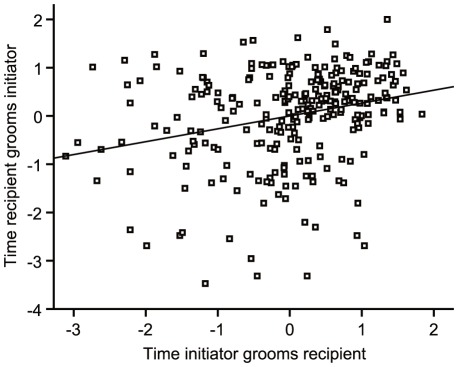
Degree of time-matching by females in reciprocal grooming bouts. The line represents complete time-matching by partners. Among reciprocated grooming bouts, recipient's grooming time as a function of initiator's grooming time for golden snub-nosed monkey (*N* = 254 bouts). Grooming durations (s) were logarithm transformed then normalized.

Analyses of all reciprocated grooming bouts suggested that the relationship between the amount of time each initiator spent grooming the recipient and the amount of time the receiver reciprocated was extremely significant (weighted least-squares regression analysis, *F*_1,102_ = 7.91, *p* = 0.0059). Both males and females demonstrated time matching reciprocated grooming bouts (Spearman correlation: *r_s_* = 0.35, *p*<0.001). The total duration of time reciprocated by the recipient was positively correlated with the total duration of time the initiator spent grooming within a bout. The time matching slopes were positive (*b* ± SE = 0.26±0.09, *β* = 0.27; Fig. 3).

### Male vs. female dyads

**Figure 4:**
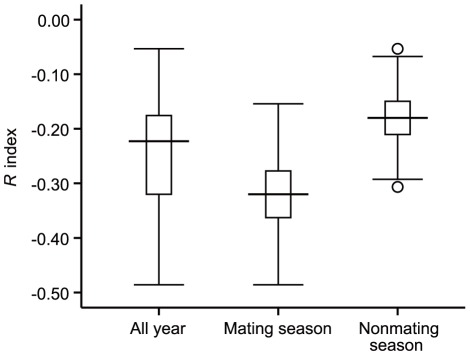
Mean ± SE (boxes) *R* indices within the male-female categories of different periods. Whiskers indicate the 95% confidence interval of the means; outliers are given as dots. The negative value of mean *R* indicates that males received more grooming from females than they gave in return (mean *R* ± SE = −0.27). Males received more grooming from females in the mating season (mean *R* ± SE = −0.32±0.01) than that in the non-mating season (mean *R* ± SE = −0.18±0.02).

A very large discrepancy in grooming time was found in male-female dyads. The value of mean *R* was negative, indicating that males received more grooming from females than they gave in return (mean *R* ± SE = −0.27±0.06; Fig. 3). There was a significant difference between the expected duration and the observed duration of grooming that each resident male reciprocated to his harem females. (*χ* ^2^ = 2505.122, *df* = 5, *p*<0.0001).The correlation between the degree of male grooming reciprocity and the number of females within each one-male unit was significantly negative (*r_s_* = −0.85, *p*<0.05; Fig. 4), which indicated that the reciprocation of grooming from males decreased with an increasing number of females in the one-male unit. In addition, there was a significant difference in grooming reciprocation from males between the mating and non-mating seasons (*t* = −2.40, *p*<0.05), with males receiving more grooming from females during the mating season (mean *R* ± SE = −0.32±0.01) than during the non-mating season (mean *R* ± SE = −0.18±0.02; Fig. 4). There was a positive relationship between the rate of copulation per day for each female and the total duration of grooming time that females invested in males during the mating season (*r* = 0.76, *p*<0.05). The rate of food- related aggressive interactions per statistical day (directed from males to females) was negatively correlated with the duration of grooming that females invested to the resident male in each one-male unit (*r* = −0.63, *p*<0.05).

### Female vs. female dyads

Contrary to male-female dyads, the dyads consisting of females showed no significant correlation between grooming reciprocity and the adult sex ratio (male∶female) within each one-male unit (*r_s_* = 0.54, *p* = 0.09). Although not statistically significant, this result suggests that an increase in the number of females might increase the odds of reciprocation. Therefore, the female-female dyad data from both the mating season and the non-mating season were analyzed separately to explore the possibility of seasonal effects. Grooming reciprocation was significantly and negatively correlated with the number of females within a one-male unit during the mating season (*r_s_* = −0.64, *p*<0.05). In the non-mating season, however, the level of reciprocation and the number of females was negatively, but not significantly, correlated (*r_s_* = −0.48, *p* = 0.08). A significant difference in grooming reciprocation during and outside the mating season was obeserved (*t* = −3.56, *p*<0.001). Approximately 11.1% of females in the study group had no young offspring and did not get pregnant during the year of study. Aggressive interactions occurred at a rate of 0.33 events per statistical day in the mating season and 0.13 outside the mating season. Aggressive interactions were thus not equally distributed during the mating season and outside the mating season (*χ* ^2^ = 21.52, *p*<0.001). The level of female-female aggression was higher in the mating season than outside of the mating season (*χ* ^2^ = 11.34, *p*<0.001).

**Figure 5:**
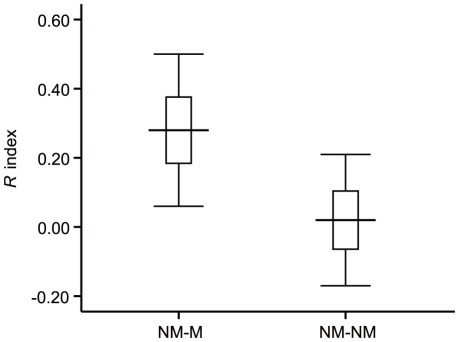
Mean ± SE (boxes) *R* indices within the two female vs. female categories. Whiskers indicate the 95% confidence interval of the means. NM-M: dyads consisting of non-mother and mothers; NM-NM: dyads consisting of non-mothers. The value of *R* index in dyads consisting of non-mother and mother was positive (mean *R* ± SE = 0.28±0.02) showing that mothers received more grooming from non-mothers than they gave in return. The dyads of non-mothers represented approximately symmetry on the amount of time they invested in grooming each other within each dyads (mean *R* ± SE = 0.01±0.09).

A strong discrepancy of grooming was found in dyads consisting of non-mothers and mothers with the *R* index being positive (mean *R* ± SE = 0.28±0.02; Fig. 5). These results suggested that the average time non-mothers spent grooming mothers was more than they spent grooming other females (paired t test: *t*_27_ = −3.01, *p* = 0.03). The mean number of infants per one-male unit was 0.43±0.12 (mean ± SD). The average number of adult females without infants per one-male unit was 2.55±1.47 (mean ± SD). The average number of juvenile females per one-male unit was 0.58±0.72 (mean±SD). A significant negative relationship was observed between non-mother to mother grooming duration and the rate of infants per female in each one-male unit (*r* = −0.62, *p*<0.05). This relationship indicated that when infants were abundant, grooming durations were shorter; when infants were scarce, grooming durations were longer.

Opposite to the non-mother vs. mother dyads, the dyads of non-mothers represented approximate symmetry on the amount of time they invested in grooming each other within each dyad (mean *R* ± SE = 0.01±0.09; Fig. 5). Grooming reciprocation between the two kinds of dyads was significantly different (*t* = 2.94, *p*<0.05).

**Figure 6:**
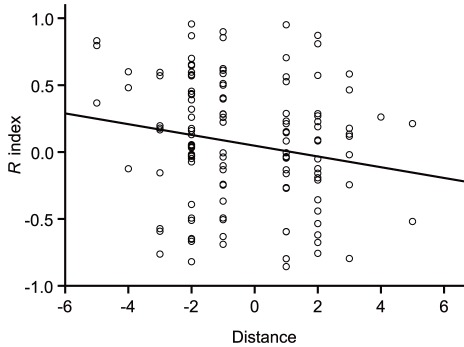
The level of grooming reciprocation was negatively correlated to the rank distance between females (*r_s_* = −0.64, *p*<0.05) . In dyads consisting of distantly ranked females, the degree of reciprocation was lower than that in dyads consisting of closely ranked females.

In dyads of high-ranking and low-ranking females, grooming was biased in favor of the former as low-ranking females groomed high-ranking females more than vice versa (mean *R* ± SE = −0.25±0.05). Moreover, social rank was one of the main factors determining the degree of grooming reciprocation (*F*_1,627_ = 4.54, *p*<0.05). The level of grooming reciprocation was negatively correlated to the rank distance between females (*r_s_* = −0.64, *p*<0.05), which meant that, in dyads consisting of distantly ranked females, the degree of reciprocation was lower than that in dyads consisting of closely ranked females (Fig. 6). There was a negative relationship between the rate of food- related aggressive interactions per statistical day and the duration of grooming that low ranking females gave to high ranking females (*r* = −0.71, *p*<0.05).

## Discussion

Among the *R. roxellana* study group, about 29.7% of all grooming bouts were reciprocated within the bout, which was an intermediate level of reciprocation when compared with other species (e.g. mangabeys: 33% [55]; chacma baboons: 31–51% [24]; bonnet macaques: 5–7%, and capuchins: 12–27% [36]). These differences may result from diversities among species or different definitions of grooming bouts [36], [61]. In addition, *R. roxellana* demonstrated time matching in grooming bouts, namely the more grooming the initiator provided to the recipient, the more likely the recipient would be to groom the initiator in return. Similar results have also been found in other studies of female primates (e.g. [24], [36], [55], [62]), suggesting that time matching may be a widespread characteristic of female-female grooming in female-bonded primate species. For *R. roxellana*, however, time matching in grooming also occured between males and females. Similarly, chimpanzees have also demonstrated time matching in male-male grooming [63]. Our results indicate that *R. roxellana* exchanged grooming for grooming and that the value of grooming may be set by the local market. In reciprocated grooming bouts, the duration of time that partners groomed each other tended to be positively correlated. However, the duration of time that the initiator groomed the recipient was only a partial predictor of the duration of time that the initiator would be groomed in return. This means that while the recipient might return the grooming favor, this, in itself, did not predict precisely how much grooming was returned. Henzi et al. [64] proposed that within-bout reciprocation was essential for the maintenance of grooming dyads over time, suggesting it was important to have the capacity to respond to grooming immediately. previous capuchin research found that grooming was balanced within dyads [2], but lopsided within bouts [36]. This means that even though the majority of grooming bouts were unidirectional, the individuals in these groups continued to groom each other over longer time spans. Thus, primates preferentially groom those individuals that groom them most. In the present study, 70.3% of grooming bouts were not reciprocated on *R. roxellana*. Within dyads, grooming in *R. roxellana* appeared mostly asymmetric and unidirectional.

Grooming asymmetries changed with local market power resulting from social status profiles that individuals displayed in social groups such as sexual and physiological differences. The majority of grooming was skewed in favor of males in bisexual dyads. Resident males distributed their grooming services to harem females of their own choice rather than distributing their services equally. These results excluded a dilution effect. Males received most grooming from females when the number of competing females was highest. Living within a group generates competition where resources are limited in space or time [65] and, as a polygynous colobine species, males are the limiting resource for female *R. roxellana*. To obtain demanded commodities, individuals need to compete with other partners and negotiate resource distribution. Adult males were generally dominant over females and directed more aggression towards females than vice versa. Females used grooming to establish a good social relationship with the central male of their one-male unit. With that, females ensured that males tolerated them in close proximity so that they could increase their frequency of mating or access to other resources such as food. Copulation opportunities for females increased with the duration of grooming time that females invested in males and if females increased the amount of grooming offered to males, they decreased the rate of food- related aggressive interactions. Sexual competition in this polygynous species presented a skew in favor of female mate competition over the one-male unit's resident male which was especially important [66]. Females with multiple competitors experienced a high level of sexual competition for accessing the single resident male. Such asymmetries in supply and demand, which result from the different number of individuals within classes, will produce grooming reciprocation asymmetries. When the supply of females in a one-male unit increased, the demand for access to the central male increased correspondingly. These increases decrease the value of female grooming, resulting in a situation where females are expected to pay more grooming for exchanges. As the number of competitors increase, females need to increase their investment in grooming the males to gain what they want from males or special seasonal resources. Thus, market powers affect the exchange of grooming in *R. roxellana*. Similar results have been found among other primate species (baboon [67]; sooty mangabeys and vervet monkeys [68]). Many females compete and occasionally obtain access to their resident male but top groomers may be given more mating priorities, leading to the maximized probability of their reproductive success. Previous studies have found that the males of many species, such as baboons [30], chimpanzees [69], lemur [70], and wild long-tailed macaques [31], use grooming as a tradable commodity, which is exchanged for access to females to gain mating opportunities. In *R. roxellana*, however, contrary to females the one-male unit resident males experience relatively little sperm competition [71]. In virtue of the social structure, the one resident male monopolizes several females, thus experiencing no within-unit sexual competition. Male *R. roxellana* exhibit extra-unit sexual behavior, choosing females from extra-unit mating [72]. Thus for resident males, there are relatively more female partners from which to choose. Hemelrijk & Luteijn [73] proposed that the degree of female grooming reciprocity should decrease with a decrease in the adult sex ratio, since competition for access to males will disrupt female relationships. In female dyads, our data partially fit this scenario. Grooming reciprocation was negatively correlated with the number of females within a one-male unit during the mating season. However, the negative correlation between these two factors was not statistically significant during the non-mating season. With female numbers in a one-male unit increasing, females face intense competition with each other, especially during the mating season [40]. It is should be noted, however, that female *R. roxellana* give birth only once every two years if the previous offspring survives to a weaning age of five or six months, or will give birth the following year if the previous offspring dies before reaching an age of six months [40]. This reproductive strategy, in a sense, weakens competitive interactions between females for access to males and ensures that female relationships are not severely disrupted even if the adult sex ratio decreases in the non-mating season. Even under strong competition, female *R. roxellana* still possess other behavioral strategies that can be utilized to access the resident male such as female dispersal and sexual interference [38], [49], [74]. Our results suggested that for *R. roxellana*, such a market may undergo seasonal fluctuations.

Grooming reciprocation may not be necessary if females exchange grooming for other services. In dyads of non-mothers vs. mothers, non-mothers groomed mothers much more than mothers groomed them in return. The lower the rate of infants per female in each one-male unit, the longer the durations of grooming that non-mothers invested in mothers. This asymmetry of grooming reciprocation may be influenced by factors that exchange grooming for infant handling, which was supported by the fact that grooming durations were shorter when infants were abundant, but were longer when infants were scarce. The number of infants per group was relatively small and since female *R. roxellana* only give birth once every two years [40], birth events could have a significant and important impact on grooming patterns. Females may become more attractive grooming partners to other females when they have young infants [30]. In other words, as long as category roles were unambiguously assigned (non-mother vs. mother) it was possible to determine that the supply of infants had a remarkable effect on the value of grooming among females in which grooming was exchanged for access to infants. Henzi & Barrett [23] showed that the amount of grooming directed towards baboon mothers was greater when there were fewer newborn infants. Some subsequent studies have shown similar results (*Macaca fascicularis*: [32]; *Cercocebus aty*: [68]), however, other species have not conformed to this theory (*Papio anubis*: [75]; *Cebus apella nigritus*: [22]). Non-mothers displayed approximate symmetry with regards to the amount of time they invested in grooming each other within each dyad. This supported the hypothesis that when no other service was being traded, grooming was exchanged for grooming, and was therefore approximately reciprocated within a dyad. Non-mothers had no other service to offer except provide grooming or food to other non-mothers. Thus they mainly used grooming in exchange for grooming.

Schino [76] proposed that a common feature of primate grooming is the possibility of an exchange of grooming for rank-related benefits. In *R. roxellana*, more grooming was given by low-ranking females than by high-ranking females. Furthermore, the degree of reciprocation was lower in dyads consisting of closely ranked females. Although female *R. roxellana* hierarchy fluctuates over time, female ranking is a linear dominance hierarchy [60]. Evidence that *R. roxellana* directed their grooming efforts up the hierarchy and that low ranking females groomed high ranking females for a longer grooming duration to reduce the rate of food- related aggression suggests that females may trade grooming for tolerance near food resources. Higher ranked females may maintain access to higher quality food patches relative to low-ranking females. The lower ranked females in a dyad pay more in terms of grooming as power differentials increase. Similar patterns of grooming have been obtained in a number of studies on Old World monkeys [29], [34], [61], [67] and other species such as cooperatively breeding carnivores [77], [78]. This implies that rank distance may have an unassailable effect on females to interchange. We speculated that the interchange of grooming for tolerance may have existed in our study group. When competition among females was intense and dominance hierarchy relationships had powerful effects on access to resources, grooming appeared to be exchanged for tolerance, resulting in a situation where grooming discrepancies were inversely related to rank distance. The value of tolerance from a high-ranking individual, which may depend on the quantity and distribution of local resources, usually varies over time and space. In situations of low level resource competition, in which dominant individuals have fewer commodities to offer subordinates, grooming asymmetries between high-ranking females and low-ranking females decreased. The greater the power differential between two partners, the greater the value of association, since females feeding in the vicinity of a higher ranking female will experience fewer displacements due to the reluctance of other animals to approach and risk aggression from the higher ranking female. It is the risk of direct attack on non-tolerated animals by a higher ranking female that animals attempt to avoid. Barrett et al. [24] reported similar findings in which grooming exchanged within female baboons was affected by the rank distance between individuals when comparing baboon troops experiencing different patterns of dominance hierarchy relationships where grooming could be traded for support or tolerance. Similar results have also been reported in baboon and lemur [67]. Not all grooming is, however, directed up the hierarchy in exchange for rank-related benefits. Contrary to our results, Leinfelder et al. [79] reported that hamadryad baboon(*Papio hamadryas*) displayed no tendency to direct grooming up the hierarchy. They concluded that hamadryad females only traded grooming for itself and showed no evidence for interchange. In addition, the majority of females did not exchange grooming for support from non-relatives [80].

### Conclusion

In the studied *R. roxellana*, grooming was asymmetric. Grooming disparities suggested that individuals spent more time investing in social relationships with more valuable partners. Power differentials that result from social status can offset strict reciprocation and influence the dynamics of grooming between individuals in non-human primate species. In *R. roxellana*, reciprocity existed, because partners firmly distributed grooming according to their social standing irrespective of whether grooming reciprocation would occur or not [61]. To understand the dynamics of grooming, both comprehensive data and appropriate analytical methods of various interactions involving grooming in primates are required. Primate inter-individual relationships between reciprocation and interchange are a complex web. Thus, a better understanding of the temporal relationships between grooming and other behavioral candidates for reciprocation/interchange will facilitate the testing of predictions based on theoretical modeling.

## References

[1] di Bitetti MS (1997). Evidence for an important social role of allogrooming in a platyrrhine primate.. Animal Behaviour.

[2] Manson JH, Rose LM, Perry S, Gros-Louis J (1999). Dynamics of female-female relationships in wild *Cebus capucinus*: data from two costa rican sites.. International Journal of Primatology.

[3] Dunbar RIM (1988). Primate social systems.

[4] Henzi SP, Barrett L (1999). The value of grooming to female primates.. Primates.

[5] Hart BL, Hart LA, Mooring MS, Olubayo R (1992). Biological basis of grooming behaviour in antelope: the body-size, vigilance and habitat principles.. Animal Behaviour.

[6] Mooring MS, McKenzie AA, Hart BL (1996). Role of sex and breeding status in grooming and total tick load of impala.. Behavioral Ecology and Sociobiology.

[7] Pérez A, Veà J (2000). Functional implications of allogrooming in *Cercocebus torquatus*.. International Journal of Primatology.

[8] Lewis RJ (2010). Grooming patterns in Verreaux's sifaka.. American Journal of Primatology.

[9] Meller RE, Keverne EB, Herbert J (1980). Behavioural and endocrine effects of naltrexone in male talapoin monkeys.. Pharmacol Biochem Behav.

[10] Keverne EB, Martensz ND, Tuite B (1989). Beta-endorphin concentrations in cerebrospinal fluid of monkeys are influenced by grooming relationships.. Psychoneuroendocrino.

[11] Boccia ML, Reite M, Laudenslager M (1989). On the physiology of grooming in a pigtail macaque.. Physiology & Behavior.

[12] Aureli F, Preston SD, de Waal F (1999). Heart rate responses to social interactions in free-moving rhesus macaques (*Macaca mulatta*): A pilot study.. Journal of Comparative Psychology.

[13] Hemelrijk CK, Ek A (1991). Reciprocity and interchange of grooming and ‘support’ in captive chimpanzees.. Animal Behaviour.

[14] Hemelrijk CK (1994). Support for being groomed in long-tailed macaques, *Macaca fascicularis*.. Animal Behaviour.

[15] Seyfarth RM, Cheney DL (1984). Grooming, alliances and reciprocal altruism in vervet monkeys.. Nature.

[16] Hamilton WD (1964). The genetical evolution of social behaviour. I & II.. Journal of Theoretical Biology.

[17] Trivers RL (1971). The evolution of reciprocal altruism.. The Quarterly review of biology.

[18] Seyfarth RM (1977). A model of social grooming among adult female monkeys.. Journal of Theoretical Biology.

[19] Schino G, Aureli F (2008). Grooming reciprocation among female primates: a meta-analysis.. Biol Lett.

[20] Noë R, Hammerstein P (1994). Biological markets: supply and demand determine the effect of partner choice in cooperation, mutualism and mating.. Behavioral Ecology and Sociobiology.

[21] Noë R, Hammerstein P (1995). Biological markets.. Trends in Ecology & Evolution.

[22] Tiddi B, Aureli F, Schino G (2010). Grooming for infant handling in tufted capuchin monkeys: a reappraisal of the primate infant market.. Animal Behaviour.

[23] Henzi SP, Barrett L (2002). Infants as a commodity in a baboon market.. Animal Behaviour.

[24] Barrett L, Henzi SP, Weingrill T, Lycett JE, Hill RA (1999). Market forces predict grooming reciprocity in female baboons.. Proceedings of the Royal Society of London Series B: Biological Sciences.

[25] Lazaro-Perea C, Arruda MF, Snowdon CT (2004). Grooming as a reward? Social function of grooming between females in cooperatively breeding marmosets.. Animal Behaviour.

[26] Payne HFP, Lawes MJ, Henzi SP (2003). Competition and the exchange of grooming among female samango monkeys (*Cercopithecus mitis erythrarchus*).. Behaviour.

[27] de Waal FBM (1997). The Chimpanzee's service economy: Food for grooming.. Evol Hum Behav.

[28] Fruteau C, Voelkl B, van Damme E, Noë R (2009). Supply and demand determine the market value of food providers in wild vervet monkeys.. Proceedings of the National Academy of Sciences.

[29] Ventura R, Majolo B, Koyama NF, Hardie S, Schino G (2006). Reciprocation and interchange in wild Japanese macaques: grooming, cofeeding, and agonistic support.. American Journal of Primatology.

[30] Barrett L, Henzi SP, Noë R, Hooff JARAM, Hammerstein P (2001). The utility of grooming in baboon troops.. Economics in nature: social dilemmas, mate choice and biological markets.

[31] Gumert MD (2007). Payment for sex in a macaque mating market.. Animal Behaviour.

[32] Gumert MD (2007). Grooming and infant handling interchange in *Macaca fascicularis*: The relationship between infant supply and grooming payment.. International Journal of Primatology.

[33] Fruteau C, van de Waal E, van Dammea E, Noë R (2011). Infant access and handling in sooty mangabeys and vervet monkeys.. Animal Behaviour.

[34] Schino G, Ventura R, Troisi A (2003). Grooming among female Japanese macaques: distinguishing between reciprocation and interchange.. Behavioral Ecology.

[35] Schino G (2007). Grooming and agonistic support: a meta-analysis of primate reciprocal altruism.. Behav Ecol.

[36] Manson JH, David NC, Silk JB, Perry S (2004). Time-matched grooming in female primates? New analyses from two species.. Animal Behaviour.

[37] Connor RC (1995). Impala allogrooming and the parcelling model of reciprocity.. Animal Behaviour.

[38] Li BG, Zhao DP (2007). Copulation behavior within one-male groups of wild *Rhinopithecus roxellana* in the Qinling Mountains of China.. Primates.

[39] Ren RM, Yan KH, Su YJ, Zhou Y, Li JJ (2000). A field study of the society of *Rhinopithecus roxellana*.

[40] Qi XG, Li BG, Ji WH (2008). Reproductive parameters of wild female *Rhinopithecus roxellana*.. American Journal of Primatology.

[41] Qi XG, Li BG, Tan CL, Gao YF (2004). Spatial structure in a Sichuan golden snub-nosed monkey (*Rhinopithecus roxellana*) group in Qinling Mountains while being no-locomotion.. Acta Zoologica Sinica.

[42] Zhang P, Watanabe K, Li BG, Tan CL (2006). Social organization of Sichuan snub-nosed monkeys (*Rhinopithecus roxellana*) in the Qinling Mountains, Central China.. Primates.

[43] Qi XG, Li BG, Garber PA, Ji WH, Watanabe K (2009). Social dynamics of the golden snub-nosed monkey (*Rhinopithecus roxellana*): female transfer and one-male unit succession.. Am J Primatol.

[44] Qi XG, Zhang P, LI BG, W K (2010). The diversity of polygynous social systems among multi-level societies in non-human primates.. Acta Theriologica Sinica.

[45] Mori U, Kawai M (1979). Individual relationship within a unit.. Ecological and Sociological studies of Gelada Baboons.

[46] Dunbar R, Dunbar P (1975). Social Dynamics of Gelada Baboons.

[47] Abeggglen JJ (1984). On socialization in hamadryas baboon.

[48] Swedell L (2002). Affiliation among female in wild hamadryas baboons (*Papio hamadryas hamadryas*).. International Journal of Primatology.

[49] Zhao DP, Ji WH, Li BG, Watanabe K (2008). Mate competition and reproductive correlates of female dispersal in a polygynous primate species (*Rhinopithecus roxellana*).. Behavioural Processes.

[50] Silk JB (1999). Why are infants so attractive to others? The form and function of infant handling in bonnet macaques.. Anim Behav.

[51] Zhao HT (2011). Study on the correlationship between female dominance hierarchy and mating behavior of Sichuan snub-nosed monkeys (*Rhinopithecus roxellana*).

[52] Li BG, Chen C, Ji WH, Ren BP (2000). Seasonal home range changes of the Sichuan snub-nosed monkey (*Rhinopithecus roxellana*) in the Qinling Mountains of China.. Folia Primatologica.

[53] Guo ST, Li BG, Watanabe K (2007). Diet and activity budget of *Rhinopithecus roxellana* in the Qinling Mountains, China.. Primates.

[54] Altmann J (1974). Observational study of behavior: sampling methods.. Behaviour.

[55] Chancellor RL, Isbell LA (2009). Female grooming markets in a population of gray-cheeked mangabeys (*Lophocebus albigena*).. Behavioral Ecology.

[56] Henzi SP, Lycett JE, Weingrill T (1997). Cohort size and the allocation of social effort by female mountain baboons.. Anim Behav.

[57] Gould W (2000). Interpreting logistic regression in all its forms.. Stata Technical Bulletin.

[58] Hemelrijk CK (1990). Models of, and tests for, reciprocity, unidirectionality and other social interaction patterns at a group level.. Animal Behaviour.

[59] Zumpe D, Michael RP (1986). Dominance index: A simple measure of relative dominance status in primates.. American Journal of Primatology.

[60] Li BG, Li HQ, Zhao DP, Zhang YH, Qi XG (2006). Study on dominance hierarchy of Sichuan snub-nosed monkeys (*Rhinopithecus roxellana*) in Qinling Mountains.. Acta Theriologica Sinica.

[61] Port M, Clough D, Kappeler PM (2009). Market effects offset the reciprocation of grooming in free-ranging redfronted lemurs, *Eulemur fulvus rufus*.. Anim Behav.

[62] Cords M (2002). Friendship among adult female blue monkeys (*Cercopithecus mitis*).. Behaviour.

[63] Newton-Fisher NE, Lee PC (2011). Grooming reciprocity in wild male chimpanzees.. Animal Behaviour.

[64] Henzi SP, Lycett JE, Piper SE (1997). Fission and troop size in a mountain baboon population.. Animal Behaviour.

[65] Janson C, Goldsmith M (1995). Predicting group size in primates: foraging costs and predation risks.. Behavioral Ecology.

[66] Smuts BB, Smuts BB, Chency DL, Seyfarth RM, Wrangham RW, Struhsaker TT (1987). Sexual competition and mate choice.. Primate societies Part III.

[67] Barrett L, Gaynor D, Henzi SP (2002). A dynamic interaction between aggression and grooming reciprocity among female chacma baboons.. Animal Behaviour.

[68] Fruteau C, Range F, Noë R (2010). Infanticide risk and infant defence in multi-male free-ranging sooty mangabeys, *Cercocebus atys*.. Behavioural Processes.

[69] Stopka P, Johnson DDP, Barrett L (2001). “Friendship” for fitness or “friendship” for friendship's sake.. Animal Behaviour.

[70] Norscia I, Antonacci D, Palagi E (2009). Mating first, mating more: biological market fluctuation in a wild prosimian.. PLoS ONE.

[71] Dixson AF (1998). Primate sexuality: comparative studies of the prosimians, monkeys, apes, and human beings.

[72] Zhao DP, Li BG, Li YH, Wada K (2005). Extra-unit sexual behaviour among wild sichuan snub-nosed monkeys (*Rhinopithecus roxellana*) in the Qinling Mountains of China.. Folia Primatologica.

[73] Hemelrijk CK, Luteijn M (1998). Philopatry, male presence and grooming reciprocation among female primates: a comparative perspective.. Behavioral Ecology and Sociobiology.

[74] Qi XG, Yang B, Garber PA, Ji WH, Watanabe K (2011). Sexual interference in the golden snub-nosed monkey (*Rhinopithecus roxellana*): a test of the sexual competition hypothesis in a polygynous species.. American Journal of Primatology.

[75] Frank RE, Silk JB (2009). Grooming exchange between mothers and non-mothers: the price of natal attraction in wild baboons (*Papio anubis*).. Behaviour.

[76] Schino G (2001). Grooming, competition and social rank among female primates: a meta-analysis.. Animal Behaviour.

[77] Kutsukake N, Clutton-Brock TH (2006). Social functions of allogrooming in cooperatively breeding meerkats.. Anim Behav.

[78] Kutsukake N, Clutton-Brock TH (2010). Grooming and the value of social relationships in cooperatively breeding meerkats.. Animal Behaviour.

[79] Leinfelder I, de Vries H, Deleu R, Nelissen M (2001). Rank and grooming reciprocity among females in a mixed-sex group of captive hamadryas baboons.. American Journal of Primatology.

[80] Silk JB (1982). Altruism among female *Macaca radiata*: explanations and analysis of patterns of grooming and coalition formation.. Behaviour.

